# Solutions for a Generalized Type of the Fokas–Lenells Equation

**DOI:** 10.1155/2024/9977603

**Published:** 2024-05-29

**Authors:** Cesar A. Gómez S.

**Affiliations:** Department of Mathematics, Universidad Nacional de Colombia, Bogotá, Colombia

## Abstract

This work investigates the following generalization of the Fokas–Lenells equation. *ıq*_*t*_+*A*(*t*)*q*_*xx*_+*B*(*t*)*q*_*xt*_+*C*(*t*)|*q*|^2^*q*+*ıD*(*t*)|*q*|^2^*q*_*x*_=*ı*[*H*(*t*)*q*_*x*_+*F*(*t*)(|*q*|^2^*q*)_*x*_+*G*(*t*)(|*q*|^2^)_*x*_*q*] which is a Schr$\overset{¨}{o}$dinger-type equation with applications in theory of communications. Here, the coefficients are variables and depend on the temporal variable *t*. The improved tanh–coth method is used to obtain exact solutions for it in a general form. If the coefficients turn constants, the equation is known as the standard Fokas–Lenells equation (FLE) which has several applications in nonlinear science. As a particular case, novel soliton solutions, chirped solutions, and the respective chirps associated with them are derived for (FLE). Also, the work explores the behaviour of the solutions when the coefficients change in time, obtaining novel structures of the solutions which help understand in a better way the phenomenon described by the (FLE). We show the graphs of some of the solutions with the aim to compare the two cases, variable and constant coefficients. Finally, some conclusions are given.

## 1. Introduction

The nonlinear partial differential equation that we have proposed for study in this work is as follows:

(1) $$\begin{array}{c} ıq_{t}+A\left(t\right)q_{xx}+B\left(t\right)q_{xt}+C\left(t\right){\left|q\right|}^{2}q+ıD\left(t\right){\left|q\right|}^{2}q_{x}=ı\left[H\left(t\right)q_{x}+F\left(t\right){\left({\left|q\right|}^{2}q\right)}_{x}+G\left(t\right){\left({\left|q\right|}^{2}\right)}_{x}q\right], \end{array}$$

where *q*=*q*(*x*, *t*) is a complex-value function that represent the wave profile, *x* is the spatial variable, *t* the temporal variable, and where the coefficients *A*, *B*, *C*, *D*, *F*, *G*, *H* are real functions depending on the variable *t*, $ı=\sqrt{-1}$. When the coefficients turn constants, equation (1) is known as the standard dimensionless Fokas–Lenells equation (FLE) with the perturbation term:

(2) $$\begin{array}{c} ıq_{t}+Aq_{xx}+Bq_{xt}+C{\left|q\right|}^{2}q+ıD{\left|q\right|}^{2}q_{x}=ı\left[Hq_{x}+F{\left({\left|q\right|}^{2}q\right)}_{x}+G{\left({\left|q\right|}^{2}\right)}_{x}q\right], \end{array}$$

whereas previously, *q*(*x*.*t*) is the unknown function of complex value, but in this case, the coefficients *A*, *B*, *C*, *D*, *F*, *G*, *H* are now constants. The standard Fokas–Lenells equation has been studied by several authors, and exact solutions have been derived using several computational methods. The model presented in this work is a generalization of the standard model, in the sense that we have considered variable coefficients (depending on the spatial variable *t*). In a previous work, (see reference [1]), the same model was considered and traveling wave solutions were derived using the same technique that we will use here, but under following condition on its coefficients: *D*=3*F*+2*G* (considered by other authors for the standard model). In the present work, we avoid this restriction; therefore, the calculations require a different analysis to solve it. As we have mentioned previously, equation (1) is a generalization of the standard model so that the solutions are different from those obtained in that case, and as we have avoided the mentioned restriction, the solutions are now compared with those obtained in the mentioned reference. Clearly, this work has relevance in physical applications, especially in the theory of communications; furthermore, from the mathematical point of view, the generalization of the models is a relevant fact.

The standard Fokas–Lenells equation (equation (2)) was proposed a few years ago as an interesting model to study the dynamics of solitons used in some applications in communications theory, and several approaches for the respective study have been used. In [2], a special ordinary differential equation is used for obtaining chirped solutions; in [3], the Lie symmetries approach join the extended *G*′/*G*-expansion method is used to obtain optical solitons; in [4], the Sine–Gordon expansion method is used to handle equation (2) and obtain exact solutions. A detailed description of equation (2) can be found in [2–4]; however, we can mention that *A* is considered as the velocity of dispersion, *B* is the velocity of dispersion space-temporal, *C* is the autophase of modulation, and *D* is the nonlinear dispersion. In the right side of equation (2), *H* is the intermodal dispersion, *F* is a perturbation term that corresponds to self-steepening effect, and *G* is a perturbation term that determines nonlinear dispersion again.

The Fokas–Lenells equation with perturbation terms [2–4], the Chen–Lee–Liu equation with perturbation terms [5], and the nonlinear Schr $\overset{¨}{o}$ dinger-type equation (6) are examples of important equations that have been used to modelling the dynamics of solitons, especially in communications theory. Using variable coefficients, we have a generalization of those models; therefore, solutions to classical models can be derived as particular cases, and additionally, we can obtain new structures for solutions related to these models. As the coefficients change in time, this fact can be helpful in understanding in a better way the dynamics of the phenomena described by the model. In the following references, we can see the study of some nonlinear equations with variable coefficients, which give us the motivation for the study of this type of models: [7–9].

To join with the several models that are appearing each day, new analytic and computational techniques are developed with the aim to handle the respective equations; for instance, the tanh–coth method [10], the Kudryashov method [11], the *G*′/*G*^2^—method [12], the Exp (−*ϕ*(*ξ*)) method [12], the improved tanh–coth method [13], and the new extended auxiliary equation method [2, 3, 6] are some of the most used computational methods. Other techniques can be found in references [14–16] and references therein. In this work, we use the improved tanh–coth method described in reference [13] to obtain solutions of equation (1) and in a particular case to equation (2), complementing in this way the set of solutions obtained, for instance in [2–4]. The improved tanh–coth method is a generalization of the extended tanh–coth method [10], and the Kudryashov method [11], as well as the G′/G-expansion method used widely in the analysis of several nonlinear equations. The method used here [13] was used previously in other works in a satisfactory way, and it is easy to implement using software such as Mathematica or Maple.

## 2. Exact Solutions

We consider the solution of equation (1) in the following form:

(3) $$\begin{array}{c} \left\{\begin{array}{c} q\left(x,t\right)=u\left(\xi \right)e^{ı\left(\Phi \left(\xi \right)+\int \rho \left(t\right)dt+\xi_{1}\right)},\\ \xi =x+\int \lambda \left(t\right)dt+\xi_{0}, \end{array}\right. \end{array}$$

being *ξ*_0_ and *ξ*_1_ arbitrary constants. Now, using (3), equation (1) converts into the following two equations that correspond to the imaginary and real parts, respectively,

(4) $$\begin{array}{c} \left\{\begin{array}{c} \left(\lambda +B\rho -H\right)u^{\prime }\left(\xi \right)+\left(2A+2B\lambda \right)u^{\prime }\left(\xi \right)\Phi^{\prime }\left(\xi \right)+B\lambda u\left(\xi \right)\Phi^{\prime \prime }\left(\xi \right)-\left(3F+2G\right)u^{2}\left(\xi \right)u^{\prime }\left(\xi \right)=0,\\ \left(-H+\lambda +B\rho \right)u\left(\xi \right)\Phi^{\prime }\left(\xi \right)-Fu^{2}\left(\xi \right)\Phi^{\prime }\left(\xi \right)+\rho u\left(\xi \right)-\left(A+B\lambda \right)u\left(\xi \right){\left(\Phi^{\prime }\left(\xi \right)\right)}^{2}-Cu^{3}\left(\xi \right)+Du^{3}\left(\xi \right)\Phi^{\prime }\left(\xi \right)=0, \end{array}\right. \end{array}$$

where ‴^″^ denotes the ordinary derivation respect to *ξ* and all coefficients of system (4) are functions depending on *t*, and by simplicity, we omit this notation here and as follows. Multiplying the first equation of system (4) with *u*′(*ξ*) and integrating with respect to *ξ*, we obtain

(5) $$\begin{array}{c} \frac{\lambda +B\rho -H}{2}u^{2}\left(\xi \right)+\left(A+B\lambda \right)u^{2}\left(\xi \right)\Phi^{\prime }\left(\xi \right)+\frac{D}{4}u^{4}\left(\xi \right)-\frac{3F+2G}{4}u^{4}\left(\xi \right)=0, \end{array}$$

and as we are interested in exact solutions, we have taken the integration constant as zero. From (5), we have

(6) $$\begin{array}{c} \Phi^{\prime }\left(\xi \right)=\frac{H-\lambda -B\rho }{2\left(A+B\lambda \right)}+\frac{3F+2G-D}{4\left(A+B\lambda \right)}u^{2}\left(\xi \right). \end{array}$$

Substituting this last expression into the second equation of (4), the system converts to

(7) $$\begin{array}{c} -4\left[H^{2}+\lambda^{2}-2B\lambda \rho -2H\left(\lambda +B\rho \right)+\rho \left(-4A+B^{2}\rho \right)\right]u\left(\xi \right)\\ -8\left[2AC+FH+2BC\lambda -F\lambda -BF\rho +2D\left(-H+\lambda +B\rho \right)\right]u^{3}\left(\xi \right)\\ -\left[7D^{2}-22DF+3F^{2}-12DG-4FG-4G^{2}\right]u^{5}\left(\xi \right)\\ -16{\left(A+B\lambda \right)}^{2}u^{\prime \prime }\left(\xi \right)=0. \end{array}$$

For solving (7), we will use the improved tanh–coth method [13, 17]. In this case, we need to make the following change of the variable:

(8) $$\begin{array}{c} u\left(\xi \right)=v{\left(\xi \right)}^{1/2}. \end{array}$$

Applying (8) to (7) and after simplifications, we have the new system:

(9) $$\begin{array}{c} -4\left[H^{2}+\lambda^{2}-2B\lambda \rho -2H\left(\lambda +B\rho \right)+\rho \left(-4A+B^{2}\rho \right)\right]v^{2}\left(\xi \right)\\ -8\left[2AC+FH+2BC\lambda -F\lambda -BF\rho +2D\left(-H+\lambda +B\rho \right)\right]v^{3}\left(\xi \right)\\ -\left[7D^{2}-22DF+3F^{2}-12DG-4FG-4G^{2}\right]v^{4}\left(\xi \right)\\ -16{\left(A+B\lambda \right)}^{2}\left(-\frac{1}{4}v^{\prime 2}\left(\xi \right)+\frac{1}{2}v\left(\xi \right)v^{\prime \prime }\left(\xi \right)\right)=0. \end{array}$$

We consider solutions to (9) as follows:

(10) $$\begin{array}{c} v\left(\xi \right)=\overset{M}{\underset{i=0}{\sum }}a_{i}\left(t\right)\phi {\left(\xi \right)}^{i}+\overset{2M}{\underset{i=M+1}{\sum }}a_{i}\left(t\right)\phi {\left(\xi \right)}^{M-i}, \end{array}$$

where *ϕ*(*ξ*) satisfies the Riccati equation

(11) $$\begin{array}{c} \phi^{\prime }\left(\xi \right)=\gamma \left(t\right)\phi^{2}\left(\xi \right)+\beta \left(t\right)\phi \left(\xi \right)+\alpha \left(t\right). \end{array}$$

Now, using (10) and balancing *v*^4^(*ξ*) with *v*(*ξ*)*v*^″^(*ξ*) in (9), we have *M*=1 so that (10) reduces to

(12) $$\begin{array}{c} v\left(\xi \right)=a_{0}\left(t\right)+a_{1}\left(t\right)\phi \left(\xi \right)+a_{2}\left(t\right)\phi^{-1}\left(\xi \right). \end{array}$$

Now, by replacing (12) into (9) and using (11), we obtain the following extensive system:

−16*a*_1_^2^*A*^2^*βγ* − 16*a*_0_*a*_1_*A*^2^*γ*^2^ − 32*a*_1_^2^*AβBγλ* − 32*a*_0_*a*_1_*ABγ*^2^*λ* − 16*a*_1_^3^*AC* − 16*a*_1_^2^*βB*^2^*γλ*^2^ − 16*a*_0_*a*_1_*B*^2^*γ*^2^*λ*^2^−16*a*_1_^3^*BCλ*+8*a*_1_^3^*BFρ* − 16*a*_1_^3^*BPρ* − 12*a*_0_*a*_1_^3^*F*^2^+16*a*_0_*a*_1_^3^*FG* − 8*a*_1_^3^*FH*+8*a*_1_^3^*Fλ*+88*a*_0_*a*_1_^3^*FP*+16*a*_0_*a*_1_^3^*G*^2^+48*a*_0_*a*_1_^3^*GP*+16*a*_1_^3^*HP* − 28*a*_0_*a*_1_^3^*P*^2^ − 16*a*_1_^3^*λP*=0.

−12*a*_1_^2^*A*^2^*γ*^2^ − 24*a*_1_^2^*ABγ*^2^*λ* − 12*a*_1_^2^*B*^2^*γ*^2^*λ*^2^ − 3*a*_1_^4^*F*^2^+4*a*_1_^4^*FG*+22*a*_1_^4^*FP*+4*a*_1_^4^*G*^2^+12*a*_1_^4^*GP* − 7*a*_1_^4^*P*^2^=0.−16*αa*_1_*a*_0_*A*^2^*γ* − 8*a*_1_*a*_0_*A*^2^*β*^2^ − 48*a*_1_*a*_2_*A*^2^*βγ* − 32*αa*_1_*a*_0_*ABγλ* − 16*a*_1_*a*_0_*Aβ*^2^*Bλ* − 96*a*_1_*a*_2_*AβBγλ* − 48*a*_1_*a*_0_^2^*AC*−48*a*_1_^2^*a*_2_*AC*+32*a*_1_*a*_0_*Aρ* − 16*αa*_1_*a*_0_*B*^2^*γλ*^2^ − 8*a*_1_*a*_0_*β*^2^*B*^2^*λ*^2^ − 48*a*_1_*a*_2_*βB*^2^*γλ*^2^ − 8*a*_1_*a*_0_*B*^2^*ρ*^2^ − 48*a*_1_*a*_0_^2^*BCλ*, −48*a*_1_^2^*a*_2_*BCλ*+24*a*_1_*a*_0_^2^*BFρ*+24*a*_1_^2^*a*_2_*BFρ*+16*a*_1_*a*_0_*BHρ*+16*a*_1_*a*_0_*Bλρ* − 48*a*_1_*a*_0_^2^*BPρ* − 48*a*_1_^2^*a*_2_*BPρ* − 12*a*_1_*a*_0_^3^*F*^2^ − 36*a*_1_^2^*a*_2_*a*_0_*F*^2^+16*a*_1_*a*_0_^3^*FG*+48*a*_1_^2^*a*_2_*a*_0_*FG* − 24*a*_1_*a*_0_^2^*FH* − 24*a*_1_^2^*a*_2_*FH*+24*a*_1_*a*_0_^2^*Fλ*+24*a*_1_^2^*a*_2_*Fλ*, +88*a*_1_*a*_0_^3^*FP*+264*a*_1_^2^*a*_2_*a*_0_*FP*+16*a*_1_*a*_0_^3^*G*^2^+48*a*_1_^2^*a*_2_*a*_0_*G*^2^+48*a*_1_*a*_0_^3^*GP*+144*a*_1_^2^*a*_2_*a*_0_*GP* − 8*a*_1_*a*_0_*H*^2^+16*a*_1_*a*_0_*Hλ*+48*a*_1_*a*_0_^2^*HP*+48*a*_1_^2^*a*_2_*HP* − 8*a*_1_*a*_0_*λ*^2^ − 28*a*_1_*a*_0_^3^*P*^2^ − 84*a*_1_^2^*a*_2_*a*_0_*P*^2^ − 48*a*_1_*a*_0_^2^*λP* − 48*a*_1_^2^*a*_2_*λP*=0.

−8*αa*_1_^2^*A*^2^*γ* − 4*a*_1_^2^*A*^2^*β*^2^ − 24*a*_0_*a*_1_*A*^2^*βγ* − 24*a*_2_*a*_1_*A*^2^*γ*^2^ − 16*αa*_1_^2^*ABγλ* − 8*a*_1_^2^*Aβ*^2^*Bλ* − 48*a*_0_*a*_1_*AβBγλ*, −48*a*_2_*a*_1_*ABγ*^2^*λ* − 48*a*_0_*a*_1_^2^*AC*+16*a*_1_^2^*Aρ* − 8*αa*_1_^2^*B*^2^*γλ*^2^ − 4*a*_1_^2^*β*^2^*B*^2^*λ*^2^ − 24*a*_0_*a*_1_*βB*^2^*γλ*^2^−24*a*_2_*a*_1_*B*^2^*γ*^2^*λ*^2^ − 4*a*_1_^2^*B*^2^*ρ*^2^ − 48*a*_0_*a*_1_^2^*BCλ*+24*a*_0_*a*_1_^2^*BFρ*+8*a*_1_^2^*BHρ*+8*a*_1_^2^*Bλρ* − 48*a*_0_*a*_1_^2^*BPρ* − 12*a*_2_*a*_1_^3^*F*^2^, −18*a*_0_^2^*a*_1_^2^*F*^2^+16*a*_2_*a*_1_^3^*FG*+24*a*_0_^2^*a*_1_^2^*FG* − 24*a*_0_*a*_1_^2^*FH*+24*a*_0_*a*_1_^2^*Fλ*+88*a*_2_*a*_1_^3^*FP*+132*a*_0_^2^*a*_1_^2^*FP*+16*a*_2_*a*_1_^3^*G*^2^+24*a*_0_^2^*a*_1_^2^*G*^2^+48*a*_2_*a*_1_^3^*GP*+72*a*_0_^2^*a*_1_^2^*GP* − 4*a*_1_^2^*H*^2^+8*a*_1_^2^*Hλ*+48*a*_0_*a*_1_^2^*HP* − 4*a*_1_^2^*λ*^2^ − 28*a*_2_*a*_1_^3^*P*^2^ − 42*a*_0_^2^*a*_1_^2^*P*^2^ − 48*a*_0_*a*_1_^2^*λP*=0.

−48*αa*_1_*a*_2_*A*^2^*β* − 16*αa*_2_*a*_0_*A*^2^*γ* − 8*a*_2_*a*_0_*A*^2^*β*^2^ − 96*αa*_1_*a*_2_*AβBλ* − 32*αa*_2_*a*_0_*ABγλ* − 16*a*_2_*a*_0_*Aβ*^2^*Bλ* − 48*a*_2_*a*_0_^2^*AC* − 48*a*_1_*a*_2_^2^*AC*+32*a*_2_*a*_0_*Aρ* − 48*αa*_1_*a*_2_*βB*^2^*λ*^2^ − 16*αa*_2_*a*_0_*B*^2^*γλ*^2^ − 8*a*_2_*a*_0_*β*^2^*B*^2^*λ*^2^, −8*a*_2_*a*_0_*B*^2^*ρ*^2^ − 48*a*_2_*a*_0_^2^*BCλ* − 48*a*_1_*a*_2_^2^*BCλ*+24*a*_2_*a*_0_^2^*BFρ*+24*a*_1_*a*_2_^2^*BFρ*+16*a*_2_*a*_0_*BHρ*+16*a*_2_*a*_0_*Bλρ*, −48*a*_2_*a*_0_^2^*BPρ* − 48*a*_1_*a*_2_^2^*BPρ* − 12*a*_2_*a*_0_^3^*F*^2^ − 36*a*_1_*a*_2_^2^*a*_0_*F*^2^+16*a*_2_*a*_0_^3^*FG*+48*a*_1_*a*_2_^2^*a*_0_*FG*, −24*a*_2_*a*_0_^2^*FH* − 24*a*_1_*a*_2_^2^*FH*+24*a*_2_*a*_0_^2^*Fλ*+24*a*_1_*a*_2_^2^*Fλ*+88*a*_2_*a*_0_^3^*FP*+264*a*_1_*a*_2_^2^*a*_0_*FP*+16*a*_2_*a*_0_^3^*G*^2^+48*a*_1_*a*_2_^2^*a*_0_*G*^2^+48*a*_2_*a*_0_^3^*GP*+144*a*_1_*a*_2_^2^*a*_0_*GP* − 8*a*_2_*a*_0_*H*^2^+16*a*_2_*a*_0_*Hλ*+48*a*_2_*a*_0_^2^*HP*+48*a*_1_*a*_2_^2^*HP* − 8*a*_2_*a*_0_*λ*^2^ − 28*a*_2_*a*_0_^3^*P*^2^ − 84*a*_1_*a*_2_^2^*a*_0_*P*^2^ − 48*a*_2_*a*_0_^2^*λP* − 48*a*_1_*a*_2_^2^*λP*=0.

−3*F*^2^*a*_0_^4^+4*G*^2^*a*_0_^4^ − 7*P*^2^*a*_0_^4^+4*FGa*_0_^4^+22*FPa*_0_^4^+12*GPa*_0_^4^ − 16*ACa*_0_^3^ − 8*FHa*_0_^3^+16*HPa*_0_^3^ − 16*BCλa*_0_^3^+8*Fλa*_0_^3^ − 16*Pλa*_0_^3^+8*BFρa*_0_^3^ − 16*BPρa*_0_^3^ − 4*H*^2^*a*_0_^2^ − 4*λ*^2^*a*_0_^2^ − 4*B*^2^*ρ*^2^*a*_0_^2^+8*Hλa*_0_^2^+16*Aρa*_0_^2^+8*BHρa*_0_^2^+8*Bλρa*_0_^2^ − 36*F*^2^*a*_1_*a*_2_*a*_0_^2^+48*G*^2^*a*_1_*a*_2_*a*_0_^2^ − 84*P*^2^*a*_1_*a*_2_*a*_0_^2^+48*FGa*_1_*a*_2_*a*_0_^2^+264*FPa*_1_*a*_2_*a*_0_^2^+144*GPa*_1_*a*_2_*a*_0_^2^ − 8*B*^2^*αβλ*^2^*a*_1_*a*_0_ − 8*A*^2^*αβa*_1_*a*_0_ − 16*ABαβλa*_1_*a*_0_ − 8*B*^2^*βγλ*^2^*a*_2_*a*_0_, $\begin{array}{c} -8A^{2}\beta \gamma a_{2}a_{0}-16AB\beta \gamma \lambda a_{2}a_{0}-96ACa_{1}a_{2}a_{0}-48FHa_{1}a_{2}a_{0}+96HPa_{1}a_{2}a_{0}-96BC\lambda a_{1}a_{2}a_{0}+48F\lambda a_{1}a_{2}a_{0}\\ -96P\lambda a_{1}a_{2}a_{0}+48BF\rho a_{1}a_{2}a_{0}-96BP\rho a_{1}a_{2}a_{0}+4A^{2}\alpha^{2}a_{1}^{2}+4B^{2}\alpha^{2}\lambda^{2}a_{1}^{2}+8AB\alpha^{2}\lambda a_{1}^{2}+ \end{array}$, $\begin{array}{c} 4A^{2}\gamma^{2}a_{2}^{2}+4B^{2}\gamma^{2}\lambda^{2}a_{2}^{2}-18F^{2}a_{1}^{2}a_{2}^{2}+24G^{2}a_{1}^{2}a_{2}^{2}-42P^{2}a_{1}^{2}a_{2}^{2}+24FGa_{1}^{2}a_{2}^{2}+132FPa_{1}^{2}a_{2}^{2}+72GPa_{1}^{2}a_{2}^{2}\\ +8AB\gamma^{2}\lambda a_{2}^{2}-8H^{2}a_{1}a_{2}-24A^{2}\beta^{2}a_{1}a_{2}-24B^{2}\beta^{2}\lambda^{2}a_{1}a_{2}-48B^{2}\alpha \gamma \lambda^{2}a_{1}a_{2}-8\lambda^{2}a_{1}a_{2}-8B^{2}\rho^{2}a_{1}a_{2}-48A^{2}\alpha \gamma a_{1}a_{2}\\ -48AB\beta^{2}\lambda a_{1}a_{2}+16H\lambda a_{1}a_{2}-96AB\alpha \gamma \lambda a_{1}a_{2}+32A\rho a_{1}a_{2}+16BH\rho a_{1}a_{2}+16B\lambda \rho a_{1}a_{2}=0. \end{array}$

−16*α*^2^*a*_0_*a*_2_*A*^2^ − 16*αa*_2_^2^*A*^2^*β* − 32*α*^2^*a*_0_*a*_2_*ABλ* − 32*αa*_2_^2^*AβBλ* − 16*a*_2_^3^*AC* − 16*α*^2^*a*_0_*a*_2_*B*^2^*λ*^2^ − 16*αa*_2_^2^*βB*^2^*λ*^2^ − 16*a*_2_^3^*BCλ*+8*a*_2_^3^*BFρ* − 16*a*_2_^3^*BPρ* − 12*a*_0_*a*_2_^3^*F*^2^+16*a*_0_*a*_2_^3^*FG* − 8*a*_2_^3^*FH*+8*a*_2_^3^*Fλ*+88*a*_0_*a*_2_^3^*FP*+16*a*_0_*a*_2_^3^*G*^2^+48*a*_0_*a*_2_^3^*GP*+16*a*_2_^3^*HP* − 28*a*_0_*a*_2_^3^*P*^2^ − 16*a*_2_^3^*λP*=0.

$\begin{array}{c} -24\alpha^{2}a_{1}a_{2}A^{2}-24\alpha a_{0}a_{2}A^{2}\beta -8\alpha a_{2}^{2}A^{2}\gamma -4a_{2}^{2}A^{2}\beta^{2}-48\alpha^{2}a_{1}a_{2}AB\lambda -48\alpha a_{0}a_{2}A\beta B\lambda \\ -16\alpha a_{2}^{2}AB\gamma \lambda -8a_{2}^{2}A\beta^{2}B\lambda -48a_{0}a_{2}^{2}AC+16a_{2}^{2}A\rho -24\alpha^{2}a_{1}a_{2}B^{2}\lambda^{2}-24\alpha a_{0}a_{2}\beta B^{2}\lambda^{2} \end{array}$ , $\begin{array}{c} -8\alpha a_{2}^{2}B^{2}\gamma \lambda^{2}-4a_{2}^{2}\beta^{2}B^{2}\lambda^{2}-4a_{2}^{2}B^{2}\rho^{2}-48a_{0}a_{2}^{2}BC\lambda +24a_{0}a_{2}^{2}BF\rho +8a_{2}^{2}BH\rho +8a_{2}^{2}B\lambda \rho \\ -48a_{0}a_{2}^{2}BP\rho -12a_{1}a_{2}^{3}F^{2}-18a_{0}^{2}a_{2}^{2}F^{2}\\ +16a_{1}a_{2}^{3}FG+24a_{0}^{2}a_{2}^{2}FG-24a_{0}a_{2}^{2}FH+24a_{0}a_{2}^{2}F\lambda +88a_{1}a_{2}^{3}\\ FP+132a_{0}^{2}a_{2}^{2}FP+16a_{1}a_{2}^{3}G^{2}+24a_{0}^{2}a_{2}^{2}G^{2}+48a_{1}a_{2}^{3}GP+72a_{0}^{2}a_{2}^{2}GP-4a_{2}^{2}H^{2}+8a_{2}^{2}H\lambda + \end{array}$, $\begin{array}{c} 48a_{0}a_{2}^{2}HP-4a_{2}^{2}\lambda^{2}-28a_{1}a_{2}^{3}P^{2}-42a_{0}^{2}a_{2}^{2}P^{2}-48a_{0}a_{2}^{2}\lambda P=0.-12\alpha^{2}a_{2}^{2}A^{2}-24\alpha^{2}a_{2}^{2}AB\lambda \\ -12\alpha^{2}a_{2}^{2}B^{2}\lambda^{2}-3a_{2}^{4}F^{2}+4a_{2}^{4}FG+22a_{2}^{4}FP+4a_{2}^{4}G^{2}+12a_{2}^{4}GP-7a_{2}^{4}P^{2}=0. \end{array}$

Using mathematical software, several solutions can be derived from the system; however, by simplicity and with the aim to illustrate the structure of the solutions, we consider only the following:

(13) $$\begin{array}{c} \left\{\begin{array}{c} a_{0}\left(t\right)=a_{2}\left(t\right)=\alpha \left(t\right)=0,\gamma \left(t\right)=-\frac{ia_{1}\sqrt{3F^{2}-4FG-22FP-4G^{2}-12GP+7P^{2}}}{\sqrt{12A^{2}+24AB\lambda +12B^{2}\lambda^{2}}},\\ \beta \left(t\right)=-\frac{2i\sqrt{3}\sqrt{{\left(A+B\lambda \right)}^{2}}}{B\left(2G+5P\right)\left(A+B\lambda \right)\left(2F+2G+P\right)\sqrt{3F^{2}-4FG-22FP-4G^{2}-12GP+7P^{2}}}\\ \left(\frac{1}{A+B\lambda }\left[{\left(F-2P\right)}^{2}\left(3F^{2}-4FG-22FP-4G^{2}-12GP+7P^{2}\right)\right.\right.\\ \left(A\left(3B^{2}C^{2}-6BC\left(F-2P\right)+3F^{2}-4FG-22FP-4G^{2}-12GP+7P^{2}\right)+\right.\\ B\left(3B^{2}C^{2}\lambda -2F\left(3BC\lambda +2GH+5HP+6\lambda P\right)+12BC\lambda P+3F^{2}\lambda \right.\\ {\left.\left.\left.-4G^{2}H-12GHP-5HP^{2}+12\lambda P^{2}\right)\right)\right]}^{1/2}\\ +BC\left(-3F^{2}+4FG+22FP+4G^{2}+12GP-7P^{2}\right)+3F^{3}-4F^{2}G-28F^{2}P-4FG^{2}\\ \left.-4FGP+51FP^{2}+8G^{2}P+24GP^{2}-14P^{3}\right)\rho \left(t\right)=\frac{1}{B^{2}\left(F-2P\right)\left(2G+5P\right)\left(2F+2G+P\right)}\\ \left(B\left(-2\left(\frac{\lambda }{A+B\lambda }\left[{\left(F-2P\right)}^{2}\left(3F^{2}-4FG-22FP-4G^{2}-12GP+7P^{2}\right)\left(A\left(3B^{2}C^{2}-6BC\left(F-2P\right)+\right.\right.\right.\right.\right.\right.\\ \left.3F^{2}-4FG-22FP-4G^{2}-12GP+7P^{2}\right)+B\left(3B^{2}C^{2}\lambda -2F\left(3BC\lambda +2GH+5HP+6\lambda P\right)+12BC\lambda P+\right.\\ {\left.\left.\left.3F^{2}\lambda -4G^{2}H-12GHP-5HP^{2}+12\lambda P^{2}\right)\right)\right]}^{1/2}-12BC\lambda P^{2}+4G^{2}P\left(H+\lambda \right)+12GP^{2}\left(H+\lambda \right)+5HP^{3}\\ \left.-19\lambda P^{3}\right)+2F^{2}\left(3BC\lambda +2G\left(H+\lambda \right)+5HP+23\lambda P\right)+F\left(-3P\left(8BC\lambda +5HP+29\lambda P\right)+4G^{2}\left(H+\lambda \right)+\right.\\ \left.\left.4GP\left(H+\lambda \right)\right)-6F^{3}\lambda \right)-2A\left(\frac{1}{A+B\lambda }\left[{\left(F-2P\right)}^{2}\left(3F^{2}-4FG-22FP-4G^{2}-12GP+7P^{2}\right)\right.\right.\\ \left(A\left(3B^{2}C^{2}-6BC\left(F-2P\right)+3F^{2}-4FG-22FP-4G^{2}-12GP+7P^{2}\right)+\right.\\ {\left.\left.B\left(3B^{2}C^{2}\lambda -2F\left(3BC\lambda +2GH+5HP+6\lambda P\right)+12BC\lambda P+3F^{2}\lambda -4G^{2}H-12GHP-5HP^{2}+12\lambda P^{2}\right)\right)\right]}^{1/2}\\ \left.\left.-3BC{\left(F-2P\right)}^{2}+3F^{3}-4F^{2}G-28F^{2}P-4FG^{2}-4FGP+51FP^{2}+8G^{2}P+24GP^{2}-14P^{3}\right)\right). \end{array}\right. \end{array}$$

The general solution of (11) is given by

(14) $$\begin{array}{c} \phi \left(\xi \right)=-\frac{\sqrt{\beta^{2}\left(t\right)-4\alpha \left(t\right)\gamma \left(t\right)}\tanh\left[1/2\sqrt{\beta^{2}\left(t\right)-4\alpha \left(t\right)\gamma \left(t\right)}\xi \right]+\beta }{2\gamma \left(t\right)},\;\beta^{2}\left(t\right)-4\alpha \left(t\right)\gamma \left(t\right)>0. \end{array}$$

Other types of solutions can be found in reference [17]. With the values given in equation (13), we have

(15) $$\begin{array}{c} \phi \left(\xi \right)=-\frac{\sqrt{\beta {\left(t\right)}^{2}}\tanh\left[1/2\sqrt{\beta {\left(t\right)}^{2}}\xi \right]+\beta \left(t\right)}{2\gamma \left(t\right)},\;\beta^{2}\left(t\right)>0, \end{array}$$

with *β*(*t*) an arbitrary function. Then, equation (12) takes the following form:

(16) $$\begin{array}{c} v\left(\xi \right)=a_{1}\left(t\right)\phi \left(\xi \right), \end{array}$$

where *ϕ*(*ξ*) is given by equation (15) and *a*_1_(*t*) is an arbitrary function. Equation (16) is the solution of (9) so that according to (8)

(17) $$\begin{array}{c} u\left(\xi \right)=v{\left(\xi \right)}^{1/2}={\left[a_{1}\left(t\right)\phi \left(\xi \right)\right]}^{1/2}, \end{array}$$

is the solution of equation (7). Finally, the solution of equation (1) corresponding to values given by (13) is obtained using (3) and (17). In this case, *λ*(*t*) is an arbitrary function and *ξ*_0_ and *ξ*_1_ constants.

If we take the values *A*=1, *B*=2, *C*=3, *D*=1, *F*=1, *G*=2, *H*=3, *λ*=1, *ξ*_0_=0, *ξ*_1_=0, and *a*_1_=1, we obtain for (12) the following expression:

(18) $$\begin{array}{c} v\left(\xi \right)=-\frac{3\left(2/3\sqrt{5}\tanh\left(1/3\sqrt{5}\left(t+x\right)\right)-2\sqrt{5}/3\right)}{2\sqrt{5}}. \end{array}$$

For (*x*, *t*) ∈ [−15, 10] × [0, 2], *v*_*A*1_, *v*_*A*2_, and *v*_*A*3_ are the dimensional graph, the contour graph, and the 2-dimensional graph, respectively for (18).

Now, for this set of values, the solution of (1), according to (3), takes the following form:

(19) $$\begin{array}{c} \left\{\begin{array}{c} q\left(x,t\right)={\left[-\frac{3\left(2/3\sqrt{5}\tanh\left(1/3\sqrt{5}\left(t+x\right)\right)-2\sqrt{5}/3\right)}{2\sqrt{5}}\right]}^{1/2}e^{ı\left(\Phi \left(\xi \right)+2t\right)},\\ \xi =x+t, \end{array}\right. \end{array}$$

where according to (6), Φ(*ξ*) satisfies

(20) $$\begin{array}{c} \Phi^{\prime }\left(\xi \right)=-\frac{1}{3}+\frac{1}{2}\left(-\frac{3\left(2/3\sqrt{5}\tanh\left(1/3\sqrt{5}\left(\xi \right)\right)-2\sqrt{5}/3\right)}{2\sqrt{5}}\right). \end{array}$$

As in the previous case, we can take the following variable coefficients: *A*=*t*, *B*=*t*^2^, *C*=*t*^3^, *D*=*t*, *F*=*t*, *G*=2*t*, *H*=3*t*, *λ*=2*t*, *ξ*_0_=0, *ξ*_1_=0, and *a*_1_=*t*, for obtaining the following expression for (12)

(21) $$\begin{array}{c} v\left(\xi \right)=\frac{1}{105t^{5}}\left\{\begin{array}{c} \left(2t^{2}+1\right)\left(10t^{7}+10t^{3}+\sqrt{5}\sqrt{-\frac{t^{6}\left(2t^{10}+t^{8}+4t^{6}+2t^{4}-61t^{2}-20\right)}{2t^{2}+1}}\right)\\ -it{\left(-t^{2}\right)}^{3/2}\sqrt{{\left(2t^{3}+t\right)}^{2}}\sqrt{\frac{{\left(10t^{7}+10t^{3}+\sqrt{5}\sqrt{-t^{6}\left(2t^{10}+t^{8}+4t^{6}+2t^{4}-61t^{2}-20\right)/2t^{2}+1}\right)}^{2}}{t^{10}}}\\ \tanh\left(\frac{\sqrt{{\left(10t^{7}+10t^{3}+\sqrt{5}\sqrt{-t^{6}\left(2t^{10}+t^{8}+4t^{6}+2t^{4}-61t^{2}-20\right)/2t^{2}+1}\right)}^{2}/t^{10}}\left(t^{2}+x\right)}{21\sqrt{5}}\right). \end{array}\right. \end{array}$$

As in the previous case, for (*x*, *t*) ∈ [−15, 10] × [0, 2], the 3D graph, the contour graph, and the 2D graph for (21) are showed by *v*_*B*1_, *v*_*B*2_, and *v*_*B*3_, respectively:

On the other hand, using (3), one solution for (1) has the form:

(22) $$\begin{array}{c} \left\{\begin{array}{c} q\left(x,t\right)={\left[v\left(\xi \right)\right]}^{1/2}e^{ı\left(\Phi \left(\xi \right)+\int \rho \left(t\right)dt\right)},\\ \xi =x+t^{2},\\ \rho \left(t\right)=\frac{-4t^{9}-2t^{7}+101t^{5}+40t^{3}+8\sqrt{5}\sqrt{t^{6}\left(61t^{2}-\left(\left(2t^{2}+1\right)\left(t^{4}+2\right)t^{4}\right)+20\right)/2t^{2}+1}t^{2}+4\sqrt{5}\sqrt{t^{6}\left(61t^{2}-\left(\left(2t^{2}+1\right)\left(t^{4}+2\right)t^{4}\right)+20\right)/2t^{2}+1}}{21t^{6}}. \end{array}\right. \end{array}$$

In this last equation, *v*(*ξ*), as in (21), and Φ(*ξ*) satisfy the following equation:

(23) $$\begin{array}{c} \Phi^{\prime }\left(\xi \right)=\frac{t-t^{2}\rho \left(t\right)}{2\left(t+2t^{3}\right)}+\frac{6t}{4\left(t+2t^{3}\right)}v\left(\xi \right). \end{array}$$

In (23), *v*(*ξ*) is given by (21) and *ρ*(*t*) appears in (22).

## 3. Discussion

**Figure 1:**
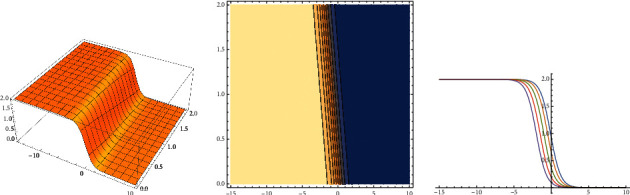
*v*_*A*1_—*v*_*A*2_—*v*_*A*3_, respectively.

**Figure 2:**
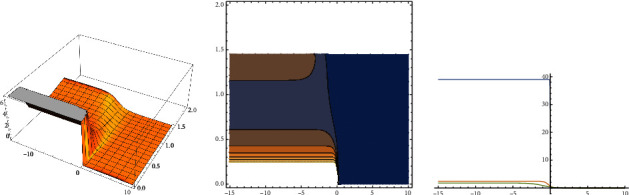
*v*_*B*1_—*v*_*B*2_—*v*_*B*3_, respectively.

The improved tanh–coth method used here can be considered as a generalization of the three well-known methods: the tanh-coth method, the Kudryashov method, and the *G*′/*G*-expansion method, and all of them were used widely to obtain exact solutions for nonlinear partial differential equations. The method can be easily implemented using mathematical software such as Mathematica or Maple. With the method used here, we have handled the extensive algebraic system (after equation (12)) to obtain solutions to construct the respective soliton solutions for equation (1). The extensive algebraic system is obtained because we have avoided the restriction on the coefficients of equation (1) mentioned in the introduction. Clearly, the model considered here is a generalization of the standard given by equation (2). With respect to the standard Fokas–Lenells equation (FLE), the authors in [2] have derived solutions using the Jacobi elliptic function. The authors in reference [1] have obtained solutions to equation (1) using a variant of the method used in [2]. The results obtained in this work are complementary to those obtained in the two mentioned references. With respect to Figures 1 and 2, we have used constant and variable coefficients, respectively, with the aim to illustrate the results. In the case of Figure 1, clearly, the solutions are in a more general form than those obtained, for instance in [1, 3].  Figure 2 shows the impact on the solutions when the coefficients change over time. In this last case, there are no blow-ups for the solutions; however, the structure of the solution changes due to the effect of the different values of the coefficients. It can be observed that the solutions are stable in the two cases, variable and constant coefficients, for the given coefficients and for the respective interval, in the case of variable coefficients. This is an important fact for physical applications.

On the other hand, clearly, we have obtained a new expression for Φ′(*ξ*) (see (20) and (23)), and with this, for each solution for the FLE, we have an associate chirp described by the relation *δw*(*x*, *t*)=−*∂*/*∂x*[Φ(*ξ*) − ∫*ρ*(*t*)*dt*]=−Φ′(*ξ*) (see [2]). This is important because not only the new chirped solutions can be used in communications, especially in the design of optic fibers, but the chirp is very important in other applications, for instance in communications of the spread spectrum, used for constructions of devices, sonar and radar (see [2] and references therein).

## 4. Conclusions

The soliton theory has practical applications in some branch of the science, and in the case of Fokas–Lenells equations, in the communications theory. The knowledge of the structure of its soliton solutions is relevant in this case. We have considered a generalization of the Fokas–Lenells equation, and we have derived exact solutions for it, which include solutions' type soliton. For that, we have used the improved tanh–coth method. It is clear that by varying the coefficients in some interval, the graphs show us that the solutions are stable, so that the model considered here gives us diverse structures for its solutions. Furthermore, the chirp associate with this model is determined by the relation *δw*(*x*, *t*)=−*∂*/*∂x*[Φ(*ξ*) − ∫*ρ*(*t*)*dt*]=−Φ′(*ξ*) (see [2]), where −Φ′(*ξ*) is given by (20) and (23). As was mentioned in the introduction, from the model considered here, new soliton solutions are derived as well as the respective chirp associated for the standard Fokas–Lenells equation FLE (constant coefficients). Our results are complementary to those obtained for instance in [3], where the authors have obtained solutions, but considering additional restrictions on the coefficients, making the initial model lose generality. Some graphics corresponding to *u*(*ξ*)^2^ (solution of (7)) have been shown to illustrate the case of constant coefficients as well as the case of variable coefficients so that using the previous relations for the chirped solutions, new expressions can be derived in the two cases, complementing the results obtained for instance in [2]. The results obtained here show that the improved tanh–coth method implemented in this work is a useful technique to handle (obtain exact solutions) to other models of nonlinear partial differential equations. In a future work, we will apply the technique to solve new nonlinear partial differential equations used to model phenomena in other fields, such as biology, applied mathematics, and other branches of physics.

## Data Availability

The data that appear in the manuscript (see equation (13)) are solutions of the system on page 4. These values have been obained using Mathematica 13.1. The graphics Figure 1 and Figure 2 have made using Mathematica 13.1.
